# Moderate Delayed Middle Cerebral Artery Vasospasm With Clinical Repercussions After the Resection of a Giant Pituitary Adenoma Using an Endonasal Endoscopic Approach: A Case Report

**DOI:** 10.7759/cureus.32415

**Published:** 2022-12-11

**Authors:** German López-Valencia, Leoncio Tovar-Romero, Juan Luis Gómez Amador

**Affiliations:** 1 Neurosurgery, Instituto Nacional de Neurología y Neurocirugía Manuel Velasco Suárez, Mexico City, MEX; 2 Neurological Surgery, Hospital General de México, Mexico City, MEX

**Keywords:** transsphenoidal resection, middle cerebral artery (mca), endonasal endoscopic surgery, nonfunctioning pituitary adenoma, vasoespasm

## Abstract

Endoscopic endonasal approach (EEA) techniques have evolved significantly in recent years, with better techniques guaranteeing better surgical results in the treatment of sellar and parasellar region pathologies. Although the complications associated with the EEA have been widely described, with cerebrospinal fluid fistulas being the most commonly reported, some rare complications, such as vasospasm after surgery in extended approaches, turn out to be poorly understood. Here, we describe a case of middle cerebral artery delayed vasospasm associated with the resection of a non-functional pituitary adenoma via an EEA.

## Introduction

The implementation of endoscopic endonasal approach (EEA) techniques for the treatment of lesions in the sellar and parasellar regions has increased considerably in recent decades, allowing a better understanding of this approach and critical technical advances in this technique. Likewise, this advance has made it possible to establish and understand better the complications associated with this approach. Vasospasm with delayed cerebral ischemia associated with EEA approaches is a rare complication occurring mainly due to the manipulation of suprasellar cistern structures. Due to the insufficient number of reports on this complication in the literature (five in total, to our knowledge), vasospasm associated with the EEA is described using the principles of vasospasm associated with non-traumatic subarachnoid hemorrhage (SAH) [1-5]. Here, we present the case of a patient with delayed middle cerebral artery (MCA) vasospasm associated with the resection of a giant pituitary adenoma using an EEA.

## Case presentation

**Figure 1:**
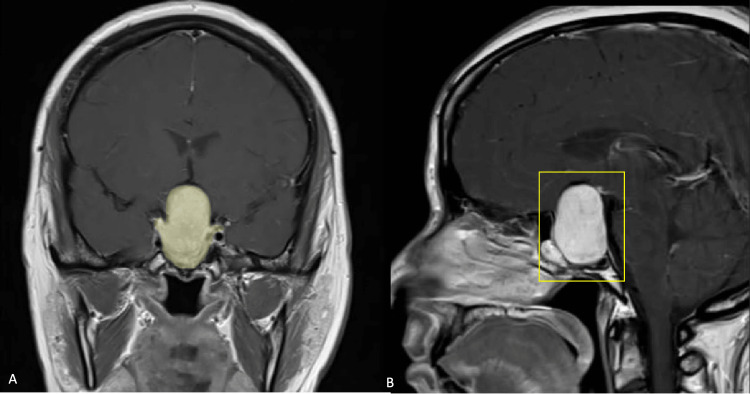
C+ MRI in coronal (A) and sagittal (B) projections showing a pituitary marcroadenoma (yellow shading) of Hardy-Wilson 2C, Knosp II classification

**Figure 2:**
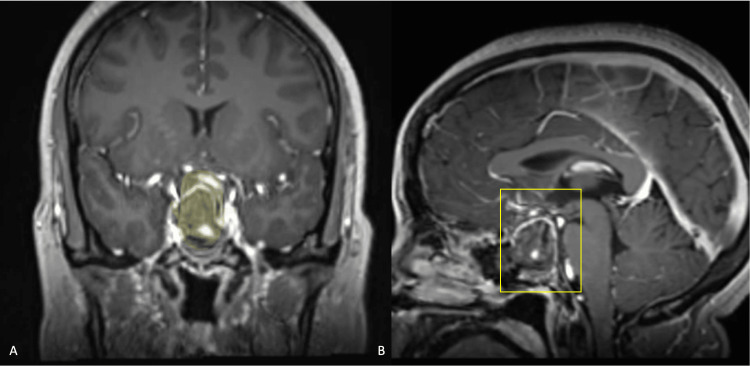
Postoperative C+ MRI in coronal (A) and sagittal (B) projections showing no residual tumor (previous tumor size is shown in yellow shading for comparison)

A 35-year-old female with no previous medical records presented to our hospital with a two-month continuous headache and progressive visual loss history. In her initial neurological examination, an obvious visual acuity loss in the right eye (20/40 using Snellen cards) and a bitemporal campimetry defect were noted. Her laboratory tests showed secondary hypothyroidism and hypocortisolism. Initial magnetic resonance imaging (MRI) showed a sellar lesion compatible with a pituitary macroadenoma of Hardy-Wilson 2C and Knosp II classification (Figure 1). A complete resection of the lesion was performed by a conventional EEA (Figure 2). No intraoperative complication occurred. Postoperative evolution was satisfactory with no early complications reported and the patient was discharged five days later.

**Figure 3:**
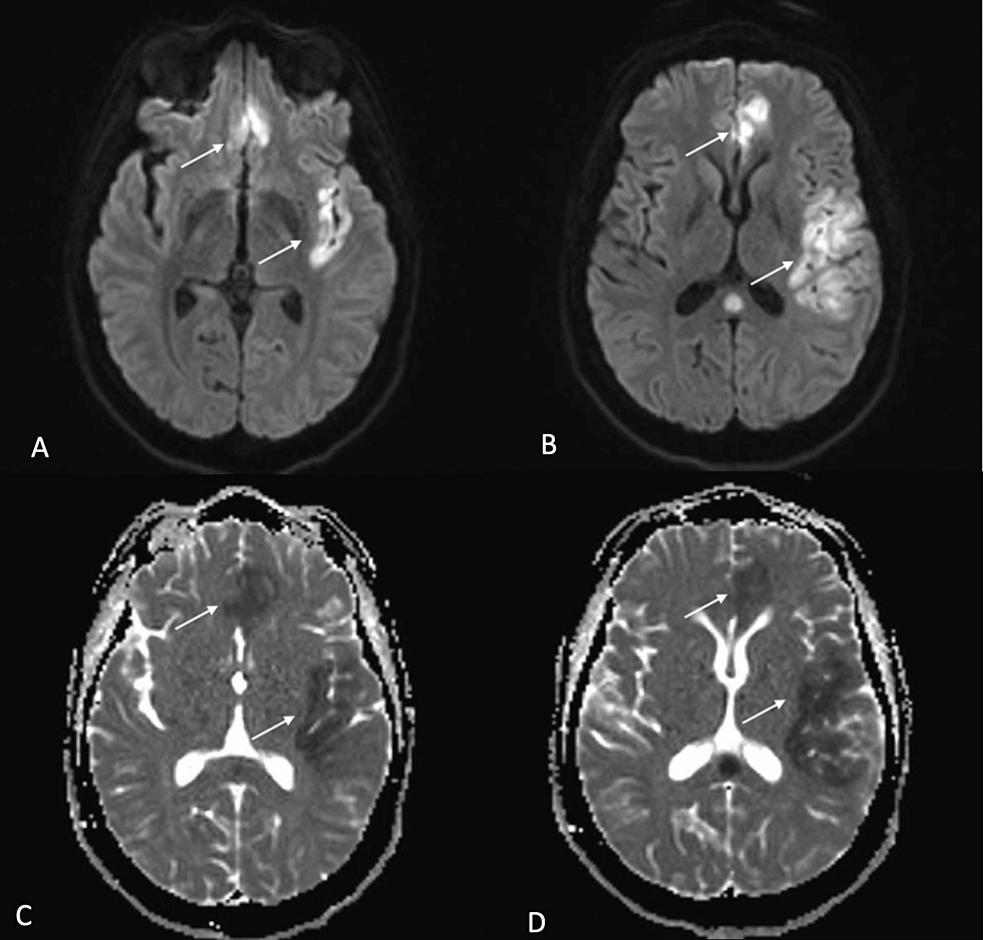
DW MRI showing diffusion restriction in the left frontal opercular cortex and precentral gyrus (A-B, white arrows), corroborated with the ADC reconstruction (B-C, white arrows) DW, diffusion-weighted; ADC, apparent diffusion coefficient

**Figure 4:**
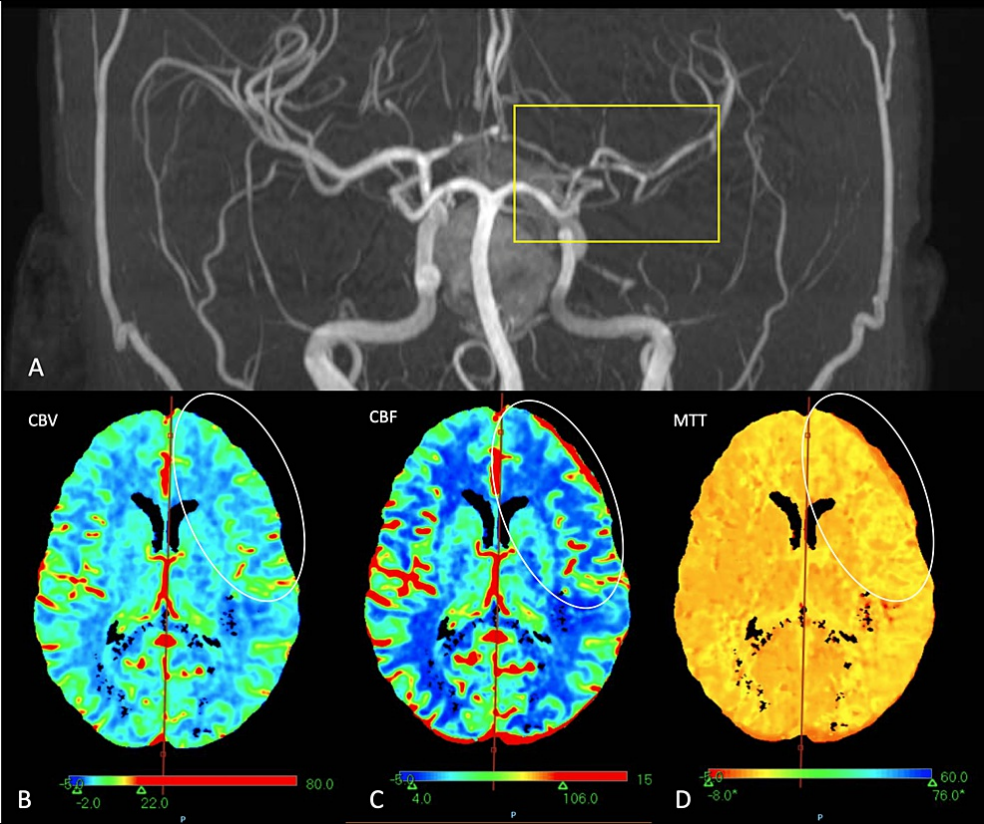
Angio-tomography showing vasospasm of the left ICA segments C5-C7, left ACA segment A1 and left MCA segment M1 (A); perfusion tomography (B-D) shows a decrease in the cerebral blood volume and blood flow velocity in the frontal opercular cortex, as well in the left precentral gyrus ICA, internal carotid artery; ACA, anterior cerebral artery; MCA, middle cerebral artery

The patient attended the emergency department 11 days after surgery reporting an acute onset of right hemiparesis and global aphasia. Diagnosis of an ischemic stroke was made with a 20-point score in the National Institutes of Health Stroke Scale (NIHSS). A cranial angio-tomography was performed finding vasospasm of the left internal carotid artery (ICA) segments C5-C7, left anterior cerebral artery (ACA) segment A1 and left MCA segment M1. The perfusion tomography showed a decrease in the cerebral blood volume (CBV) and blood flow velocity (CBF) in the frontal opercular cortex as well as in the left precentral gyrus with an Alberta Stroke Program Early CT Score (ASPECTS) score of 7 (Figure 2). It was corroborated as an area of infarction using MRI with a calculated core of 18.2 cc and penumbra of 46.1 cc (Figures 3-4). We initiated conventional vasospasm management immediately after this finding following the current American Heart Association (AHA) guidelines.

Intracranial Doppler ultrasonography (USG) was performed showing increased flow velocities in the left ICA and MCA confirming the diagnosis of moderate vasospasm with delayed cerebral ischemia. The patient was admitted to the ICU and showed clinical improvement in motor and language deficits, being discharged seven days later with a modified Rankin score of 1. The follow-up angio-CT showed resolution of the vasospasm a month after discharge.

## Discussion

The pathophysiology of vasospasm and associated delayed cerebral ischemia are well studied and are known to be an important cause of morbidity and mortality in aneurysmal SAH [6]. Various pathophysiological models have been proposed to explain vasospasm secondary to EEA surgery [5]. One of the first models proposed blood leakage into the subarachnoid space. Here hemoglobin products, such as oxyhemoglobin and methemoglobin, interact with the vascular wall causing endothelial dysfunction with decreased nitric oxide production leading to sustained vasoconstriction and subsequent ischemia [7-10]. In the particular case of EEA, the arachnoid opening with the dissemination of blood products towards the cerebral cisterns would explain the presence of vasospasm, taking into consideration that it would be expected to affect predominantly the anterior circulation for continuity. Another observation described is that the arteries enclosed by the tumor are more prone to develop vasospasm [11]. It is possible that compression and the local effect of tumor factors over the vessel wall can also cause dysfunction of the vascular tone regulation.

Another theory proposed that in large-volume tumors with hypothalamic involvement, dysfunction or direct compression damage to the hypothalamus could cause a transient catecholaminergic response with subsequent production of spasmogenic substances (e.g. endothelin or angiotensin); likewise, the presence of polyuria due to the manipulation of the hypothalamus or pituitary stalk with subsequent hypovolemia would contribute to the establishment of ischemic damage secondary to vasospasm [12-15].

In our case, the patient presented a favorable early evolution without any of the previously mentioned factors explaining the development of vasospasm and cerebral infarction; in addition, the event occurred 11 days after surgery, while in other reports, the average time to the onset of vasospasm after transsphenoidal surgery was 8 days [5,16-17]. Only one case has been reported with late vasospasm, 11 days after surgery, which was associated with meningitis due to concomitant cerebrospinal fluid (CSF) fistula, which was not found in the present case [1].

In the cases reported in the literature, the anterior circulation was mainly affected, particularly, the ACA, followed by the proximal MCA and the supraclinoid ICA; posterior circulation vasospasm was reported less frequently (proximal ACP and basilar artery) [1-5]. In the present case, the involvement of multiple anterior circulation vessels and the ICA was found. The “selective” involvement of the supraclinoid ICA and the proximal anterior circulation could be explained by the proximity of the vessels to the surgical field and the greater risk of manipulation during the procedure.

Due to the low number of cases reported, there is currently no management consensus for the treatment or prevention of vasospasm in such cases; hence, the established management guidelines for vasospasm in aneurysmal SAH must be used [18]. In this context, using trans-cranial Doppler and cranial angio-tomography can be useful in monitoring select cases with large-volume tumors with important supra-sellar involvement, vascular enclosure by the tumor, vessel manipulation and/or arachnoid aperture during surgery, as well as post-surgical complications (e.g., CSF fistula or infection), with prospective controlled studies required to determine its true usefulness [19].

The standard management of these cases should include the use of oral nimodipine as a calcium antagonist and controlled hypertension to improve cerebral perfusion [18]. The duration of this treatment depends on the severity of the condition, and it is recommended to continue nimodipine for at least 21 days after the vasospasm has been established [20]. Other treatment options such as chemical or balloon angioplasty must be individualized, requiring controlled studies to determine their efficacy and safety in these cases [5]. Prophylaxis with nimodipine cannot be recommended due to lack of evidence [20].

## Conclusions

EEA-associated vasospasm continues to be an uncommon and poorly understood entity that can have severe repercussions in postoperative patients, even in late stages, with considerable morbidity. In our case, the late presentation of this complication was a very rare finding, which is why we recommend a close follow-up of these patients during the first three weeks after surgery. Risk factors associated with surgery, as well as vasospasm predisposition post-surgery, must be detected in a timely manner to allow for adequate treatment in this select group of patients. More studies are required to establish a specific treatment plan for this complication. For now, the management guidelines established for aneurysmal SAH should be used. In our case, the early initiation of vasospasm treatment granted an excellent clinical outcome, achieving an almost total recovery of the patient.
